# Significance of *FSHR* and *LHCGR* gene polymorphisms on clinical outcomes in gonadotropin-releasing hormone antagonist protocol with freeze-all strategy: A case-control study

**DOI:** 10.18502/ijrm.v22i7.16962

**Published:** 2024-09-12

**Authors:** Jayesh Amin, Naga Sandhya Alle, Ami Patel, Bansi Prajapathi, Paresh Makwana, Jaya Prakash, Kota Murali Krishna

**Affiliations:** ^1^Wings IVF Women's Hospital, Ahmedabad, Gujarat, India.; ^2^Life Fertility and Research Center, Collector Office Jn, Maharanipeta, Visakhapatnam, Andhra Pradesh, India.

**Keywords:** LHCGR, FSHR, Polymorphism.

## Abstract

**Background:**

Follicle-stimulating hormone receptor (*FSHR*) and luteinizing hormone/choriogonadotropin receptor (*LHCGR*) are integral to ovarian function, facilitating follicle development and maturation through their respective hormonal interactions. The influence of receptor polymorphisms on the outcomes of freeze-all cycles remains unclear.

**Objective:**

This study investigates the impact of *FSHR* *N680S* and *LHCGR* *N312S* polymorphisms on clinical outcomes in freeze-all cycles.

**Materials and Methods:**

Women undergoing controlled ovarian stimulation for assisted reproductive technology participated in this study. They were administered a gonadotropin-releasing hormone antagonist protocol, with recombinant follicle-stimulating hormone (rFSH) dosages adjusted according to age, body mass index, antral follicle count, and individual hormonal responses. Additionally, human menopausal gonadotropin dosages were tailored based on the *LHCGR* *N312S* genetic variant.

**Results:**

Analysis revealed no significant differences in age, body mass index, antral follicle count, or marital status across the genotypes of *FSHR* *N680S* and *LHCGR* *N312S*. However, notable differences were observed in the rFSH dosage required daily and in total among the FSHR polymorphism genotypes. Genotypes of the *LHCGR* polymorphism correlated with fewer stimulation days. A significant interaction was observed between the 2 polymorphisms concerning total rFSH dosage.

**Conclusion:**

The presence of serine in the *FSHR* polymorphism was associated with higher rFSH dosage requirements. Both *FSHR* *N680S* and *LHCGR* *N312S* polymorphisms significantly influenced clinical pregnancy and live birth outcomes in freeze-all cycles, underscoring the potential of a pharmacogenomic approach to optimize hormone supplementation in controlled ovarian stimulation protocols during assisted reproductive technology treatments.

## 1. Introduction

Infertility/subfertility is a significant concern worldwide for couples planning to have a child. It ranks the fifth highest global problem among all other health conditions (1). The prevalence of infertility is on the rise due to various factors, including the prolonged age of a couple to plan for a pregnancy, heightened stress levels, increased exposure to various environmental pollutants, and various health conditions resulting from unhealthy lifestyles (2). Fortunately, the assisted reproductive technique is critical for an infertile couple planning for parenthood. The success of these techniques depends on the specific protocol followed, whether it be antagonist or agonist, as well as optimizing the dosage of follicle-stimulating hormone (FSH) and luteinizing hormone (LH) used for stimulation (3, 4). In recent years, there have been groundbreaking studies suggesting a pharmacogenomic approach to trailer stimulation protocols based on the genetic makeup of infertile women. This personalized approach aims to enhance the success rate of assisted reproductive technology (ART) (5–10).

The gonadotropin-releasing hormone plays a crucial role in regulating 2 essential hormones: FSH and LH (11). These 2 hormones apply themselves on their respective granulosa and theca cells through their receptors, namely follicle-stimulating hormone receptor (*FSHR*) and luteinizing hormone/choriogonadotropin receptor (*LHCGR*) (12) and help in the proliferation and maturation of follicles (13). The *FSHR* gene is present on chromosome 2, containing 10 exons; it has a common polymorphism on exon-10 known as N680S (rs6166 G > A), located in the intracellular domain. It encodes asparagine and serine amino acids, with the AAT codon coding for asparagine and the AGT codon coding for serine (14). Previous reports indicate that in regular in-vitro fertilization (IVF) cycles, individuals with *FSHR680* homozygous SS exhibit higher sensitivity to FSH, elevated basal FSH, reduced estradiol production, and increased clinical pregnancy rates compared to individuals with other *FSHR680* genotypes (14, 15).

The *LHCGR* gene contains 11 exons on chromosome 2 at cytogenic band 2p16.3 near the *FSHR* gene (7). Among the various polymorphisms identified in the *LHCGR* gene, the N312S polymorphism (rs2293275) found in exon 10 was widely studied (5, 7). N312S polymorphism substitutes asparagine with serine amino acid; the AAT codon codes the asparagine, and the AGT codon codes the serine.

Various research groups have investigated the impact of the *LHCGRN312S* polymorphic variant on controlled ovarian stimulation protocols (5, 7, 14). Previous studies have consistently shown that individuals homozygous to the serine allele have a higher chance of getting pregnant in regular IVF cycles (7, 14). Moreover, previous findings by Ga et al., showed that the homozygous serine group required higher doses of recombinant human luteinizing hormone compared to other genotypes in agonist protocol combined with a regular embryo transfer cycle (7).

To date, no research study has investigated the comparative clinical outcomes of recombinant follicle-stimulating hormone (rFSH) and human menopausal gonadotropin (hMG), and their respective combinations with gonadotropin-releasing hormone antagonistic protocol based on *FSHR* and *LHCGR* polymorphism, specifically in freeze-all embryo transfer cycles. In the current study, the objective was to assess the impact of *FSHR680* and *LHCGR312* polymorphisms on the clinical pregnancy rate and live birth rate in freeze-all cycles where the influence of hormonal stimulation on endometrial receptivity is absent due to superovulation by gonadotropins (FSH, LH).

## 2. Materials and Methods

### Study design

This observational study was conducted at the Wings IVF Center, Ahmedabad, India, from March 2019 to April 2022. During this period, data collection and participant recruitment were done, utilizing the MediTEX IVF software, Regensburg, Germany for data retrieval.

A total of 421 women initially participated in the study. Inclusion criteria: age between 25 and 40 yr, a body mass index (BMI) of less than 30 kg/m^2^, a regular menstrual cycle lasting between 21 and 35 days, presence of bilateral ovaries, non-smoking status, and a quantifiable antral follicle count (AFC).

Exclusion criteria: Participants were excluded if they were recipients of oocyte donation, had a history of pelvic inflammatory disease, possessed only one ovary, had severe endometriosis, or showed serum anti-Müllerian hormone (AMH) levels below 1 ng/mL. Additionally, exclusion criteria extended to men with significant male factor infertility (defined as oligospermia with a sperm count below 2 million/ml or azoospermia), and couples who did not complete the follow-up.

Following the application of these criteria, the study was finalized with 306 women who met all the requirements necessary for inclusion in the research analysis.

### Sample size estimation 

The sample size was determined based on a power analysis. The criteria used were effect size of f = 0.25, a significance level of 0.05, and a desired power of 0.80 (indicating an 80% chance of detecting a true effect). This analysis indicates the necessity of a total 84 participants.

In our study, the effect size for the *FSHR680* polymorphism was found to be 0.36. Given this effect size, 3 groups in the variable, a minimum sample size of 71 participants per group, and a significance level of 0.05, the power for detecting differences in total rFSH dose using one-way ANOVA was calculated to be 0.99. For *LHCGR312* and total gonadotropins, the power was calculated to be 0.86 with an effect size of 0.34 and a minimum sample size of 33 participants per group.

### Treatment protocol

Before initiating ovarian stimulation, all participants in the study were genotyped for FSH receptor (*FSHR680*) and LH/CG receptor (*LHCGR312*) polymorphisms. Stimulation commenced on either day 2 or 3 of the menstrual cycle using either rFSH alone or in combination with hMG. Dosages were initially set based on each woman's age, BMI, and AFC, with subsequent adjustments made between 150 IU and 450 IU of rFSH depending on individual responses assessed by transvaginal ultrasound scans of follicular size and serum estradiol levels, maintained below 450 pg/ml. While rFSH doses were not altered due to FSHR polymorphism results, women homozygous for *LHCGR312S* received an additional 75 IU of hMG from the first day of treatment. The protocol was set down by administering 10,000 IU of human chorionic gonadotropin as a trigger between days 10 and 12, based on estradiol levels and follicle size, followed by oocyte retrieval 36 hr later under transvaginal ultrasonography guidance.

### Genotyping of *FSHR* and *LHCGR*

In brief, the genomic DNA from peripheral leukocytes was retrieved to determine the single nucleotide polymorphisms at amino acid position 680 (Asp680Ser) in the *FSHR* gene and amino acid position 312 (N312S) in the *LHCGR* gene. The *LHCGR* and *FSHR* genes were amplified using high-fidelity PCR in a total volume of 25 µL containing 1 µL of the forward primer (5 ' -ACGCACAGTCAGGTTTAGCC-3 ' for *LHCGR* and 5 ' -CTCCTGTGCCAACCCCTTC-3 ' for *FSHR* gene) and 1 µL of the reverse primer (5 ' -AACAGCTCCGTAACCAAG-3 ' for *LHCGR* and 5 ' -TTAGATGAAATGTGTAGAAGCACTG-3 ' for *FSHR* gene) along with 15 µL PCR master mix (Takara, USA) and 100 ng template DNA. The final volume was adjusted by using dH ${\;}_{2}$ O. DNA was denatured for 10 min at 95 C before the start of the amplification program. This was followed by 37 cycles of amplification, each of which included denaturation at 95 C for 1 min, annealing at 60 C for 30 sec, and elongation at 72 C for 3 min. The final elongation was carried out for 7 min at 72 C. The PCR product was purified and checked using 2% agarose gel before being directly sequenced on an 8-capillary applied biosystems sequencing apparatus (Applied Biosystems, USA). An advanced Big Dye Terminator 3.1V was utilized for sequencing. The collected data were then compared to the reference gene in the NCBI database to analyze the polymorphism.

### Ethical considerations

Participants or their immediate relatives (in case of uneducated women who have difficulty in understanding scientific language) are provided informed consent to participate in the study. The study was approved by the Institutional Ethics Committee of Wings IVF Center, Ahmedabad, India (Code: 2019/003/31C/NSA). Ethical considerations were followed as per the Indian Council of Medical Research guidelines.

### Statistical analysis

The statistical analysis of the data was performed using R Studio version 3.6.4. Mean and standard deviation values were calculated for numerical variables like age, BMI, AFC, marital life, daily rFSH dose, total rFSH, total hMG, stimulation days, and embryology parameters (no. of oocytes, metaphase [M] 2), M1, germinal vesicles (GV), and atretic oocytes, no. of blastocysts) among genotypes and between genotypes of 2 polymorphisms. The p-values were calculated using one-way ANOVA and two-way ANOVA. For categorical variables like type of infertility, clinical pregnancy rate, and live birth rate, percentages were calculated, and Chi-square was used to calculate the p-value. P-value < 0.05 was considered statistically significant.

## 3. Results

### Genotypic distribution

The study includes 306 participants who underwent IVF treatment and received COS protocol based on functional, hormonal, and genetic biomarkers. The AA (NN) genotype distribution was 28.76% (n = 88), GA (NS) heterozygous was 48.04% (n = 147), and GG (SS) homozygous was 23.20% (n = 71) with respect to *FSHRN680S* polymorphism. For *LHCGRN312S* polymorphism, the AA (NN) genotype distribution was 10.78% (n = 33), AG (NS) heterozygous was 48.37% (n = 148), and GG (SS) homozygous was 40.85% (n = 125) (Table I). For *FSHR680* polymorphism, the allele frequency of the G allele in the South Asian population was 48%, and that of the A allele was 52% (rs6166 RefSNP Report - dbSNP - NCBI [nih.gov], and in the current study, the frequency of the G allele was 46%, and that of the A allele was 54%. The allele frequency of the A allele in the South Asian population was 39%. The G allele was 61% for *LHCGR* polymorphism from the worldwide database (rs2293275 RefSNP Report - dbSNP - NCBI [nih.gov], and the data from the present study have shown the frequency of the A allele was 21%, and the G allele was 79% for *LHCGR312 *polymorphism (Table I).

### FSHRN680S

The mean age, AFC, BMI, and marital life (yr) of study participants with *FSHR* polymorphism were similar, with no significant difference, among the 3 genotypes (NN, NS, and SS). No association was found for the type of infertility. Women with homozygous NN and heterozygous NS received a similar dose of mean daily rFSH compared to the homozygous SS group. A significant difference was observed in rFSH dose (I.U.) received per day (227.30 ± 19.2, 225.00 ± 14, and 284.50 ± 31.70 respectively, p ≤ 0.0001) and total mean rFSH dose (2786.44 ± 456.92, 2724.84 ± 410.40 and 3565.38 ± 556.91 respectively) with p ≤ 0.0001 among *FSHRN680S* genotypes (NN, NS, and SS).

The mean dose of rhMG and the number of days antagonists were administered were similar across all genotypes. Although not statistically significant, the SS group required slightly more days for ovarian stimulation (12.41 ± 1.60) and had higher mean numbers of oocytes (15.12 ± 9.70) and M2 oocytes (10.42 ± 7.06) compared to the NN and NS groups. An increasing trend was observed in the number of blastocysts formed and vitrified in the SS group, but these differences were not significant.

The SS genotype group had higher implantation (47.87%), clinical pregnancy (71.83%), and live birth rates (66.20%) compared to the NN and NS groups, but these differences were not statistically significant. Although there were no statistically significant differences in the no. of embryos transferred, an increasing trend was observed in the clinical pregnancy and live birth rates, with insignificant p-values (0.15 and 0.25), respectively (Table I).

### LHCGRN312S

Similar to *FSHRN680S* polymorphism, no significant difference was observed in the mean female age, AFC, BMI, and marital life among the 3 genotypes of *LHCGRN312S* polymorphism. An association was found between the type of infertility and the N312S genotype; the number of women with primary infertility was higher in the SS genotype among the *LHCGRN312S* genotypes (69.70%, 77.03%, 85.60%, p = 0.04).

No significant difference was observed in the daily rFSH dose among the genotypes. In the present study, the homozygous NN group required a higher total rFSH dose compared to *LHCGR312* NS and SS. In *LHCGRN312S* polymorphism, subjects in the SS group received 75 units of rhMG from day 1. The mean total number of days required for rFSH stimulation in women of the NN genotype group is significantly more (12.64 ± 1.42) when compared to women who received rFSH in NS (12.03 ± 1.42) and SS (11.99 ± 1.30) genotypes with p = 0.048.

No significant difference was observed in the mean no. of oocytes retrieved (14.78 ± 8.87) and M2 oocytes (9.98 ± 6.53) among 3 genotypes of *LHCGRN312S*. However, the mean number of oocytes retrieved in women of the NN genotype (15.87 ± 9.90) was higher compared to NS (14.47 ± 8.05), and SS (14.84 ± 9.72) women. No difference was observed in the number of M1, GV, and atretic oocytes obtained. No statistically significant difference was observed in the number and quality of blastocysts formed or vitrified among genotypes of *LHCGRN312S* polymorphism.

No significant difference was observed in the implantation rates. Also, no significant difference was observed in the clinical pregnancy rate and live birth rate among *LHCGRN312S* genotypes in freeze-all cycles (Table I).

### Combined assessment of *FSHR* and *LHCGR* on clinical outcomes

The combined polymorphism analysis of *FSHR* and *LHCGR* showed significant interaction concerning the total rFSH dose given to study participants in the controlled ovarian stimulation protocol. The higher dose requirement of total rFSH was observed when *LHCGR312* is combined with genotypes of *FSHR680* (NN: NN-2758 ± 520, NN: NS-3031 ± 764, NN: SS-4013 ± 391; NS: NN-2700 ± 333, NS: NS-2713 ± 389, NS: SS-3610 ± 611; SS: NN-2829 ± 508, SS: NS-2672 ± 324, SS: SS-3402 ± 497), showing a strong association between these 2 polymorphisms in total rFSH dose required for controlled ovarian stimulation (p = 0.02). 

Though statistically not significant, the mean number of days of antagonist supplemented was on the higher side to downregulate the pituitary gland during COS when *LHCGR* genotypes were combined with *FSHR* genotypes (NN: NN-5.53 ± 0.87, NN: NS-5.75 ± 0.75, NN: NS-6.00 ± 0.75; NS: NN-5.57 ± 0.70, NS: NS-5.59 ± 0.86, NS: NS-5.61 ± 0.98; SS: NN-5.38 ± 0.88, SS: NS-5.52 ± 0.70, SS: SS-5.53 ± 0.91). The increasing trend in the mean no. of ovarian stimulation days was observed when *LHCGR312* NN and NS compared with *FSHR* genotypes, but no such pattern was observed when *LHCGR312* SS compared with *FSHR* genotypes.

The mean no. of GV and atretic oocytes were increased in the combined assessment of *LHCGR312 *(NN and SS) and *FSHR680* genotypes (NN: NS: SS); however, no such pattern was observed with the *LHCGR312* NS group.

The mean number of Grade-I blastocysts formed and vitrified increased when *LHCGR312 *NS and SS were combined with *FSHR680* genotypes. However, these observations were not identified with *LHCGR312* NN.

Though statistically insignificant, a vital interaction was found concerning the clinical pregnancy rate (p = 0.2) and live birth rate (p = 0.25) between the 2 polymorphisms. The present data shows that increased S alleles in *FSHR680* and *LHCGR312* gene polymorphisms were associated with increased clinical outcomes. The observed upward change in clinical pregnancy rate and the live birth rate was not consistent when *LHCGR312* NN was combined with genotypes of *FSHR680* (NN: NS: SS). However, a consistent increase was observed clinical pregnancy rate and live birth rate (Table II) when *LHCGR312* NS and SS were combined with genotypes of *FSHR680*.

No statistically significant interaction or specific upward or down trend was observed between polymorphisms concerning female age, marital life, BMI, type of infertility, mean no. of AFC, IVF failures, the daily dose of rFSH, total hMG dose, mean no. of oocytes, M2, M1, rate of fertilization, mean no. blastocysts formed, and Grade-II and III blastocysts and implantation rate among all the genotype combinations.

**Table 1:** One way ANOVA for clinical and IVF outcomes with respect to *FSHR680* and *LHCGR312* variants


	***FSHR***				***LHCGR***			
**Variables**	**N/N (n = 88)**	**N/S (n = 147)**	**S/S (n = 71)**	**P-value**	**N/N (n = 33)**	**N/S (n = 148)**	**S/S (n = 125)**	**P-value**
	Female age (yr)*	30.10 ± 2.59	29.95 ± 2.66	30.46 ± 2.93	0.42	29.91 ± 2.82	30.32 ± 2.65	29.93 ± 2.75	0.44
Marital life (yr)*	5.12 ± 2.50	5.76 ± 2.68	5.76 ± 3.36	0.22	5.06 ± 2.20	5.72 ± 2.89	5.54 ± 2.85	0.46
BMI (kg/m^2^)*	25.33 ± 1.27	25.63 ± 1.39	25.58 ± 1.12	0.22	25.61 ± 1.56	25.41 ± 1.27	25.65 ± 1.26	0.34
Infertility primary**	75 (85.23)	113 (76.87)	56 (78.87)		23 (69.70)	114 (77.03%)	107 (85.60)	
Infertility secondary**	13 (14.77)	34 (23.13)	15 (21.13)	0.29	10 (30.30)	34 (22.97%)	18 (14.40)	0.06
AFC*	17.57 ± 7.38	19.12 ± 9.44	19 ± 9.93		0.41	19.30 ± 9.22	18.24 ± 8.30		18.94 ± 9.80	0.74
IVF failures*	2.28 ± 1.30	1.70 ± 0.86	1.69 ± 0.94	0.25	1.4 ± 0.52	2.0 ± 1.8	2.2 ± 0.99	0.18
rFSH dose/day (IU)*	227.3 ± 19.2	225.00 ± 14	284.50 ± 31.70	< 0.001	237.9 ± 33.7	237.5 ± 30.9	242.6 ± 33.5	0.42
Total rFSH dose (IU)*	2770 ± 451	2725 ± 418.40	3562 ± 566	< 0.001	3161 ± 768	2898 ± 567	2912 ± 527	0.06
Total rHMG dose (IU) (n = 125)*	919.64 ± 119.52	880.88 ± 73.24	892.19 ± 1	0.16	0.00 ± 0.00	0.00 ± 0.00	880 ± 97.67	NA
Total gonadotropin (IU)*	3209.09 ± 720.46	3030.27 ± 577.13	3964.08 ± 640.94	< 0.001	3161 ± 768	2898 ± 567	3808.40 ± 584.46	< 0.001
Antagonist (mean No. of days)*	5.47 ± 0.81	5.58 ± 0.80	5.62 ± 0.93	0.51	5.72 ± 0.80	5.59 ± 0.85	5.48 ± 0.81	0.25
Mean No. of stimulation days*	12.05 ± 1.34	11.95 ± 1.30	12.41 ± 1.60	0.07	12.64 ± 1.48	12.03 ± 1.43	11.99 ± 1.30	0.048
No. of oocytes*	13.63 ± 7.37	15.29 ± 9.25	15.12 ± 9.70	0.35	15.87 ± 9.90	14.47 ± 8.05	14.84 ± 9.72	0.73
Metaphase-II*	9.16 ± 5.29	10.27 ± 6.94	10.42 ± 7.06	0.37	10.39 ± 6.41	9.75 ± 5.94	10.14 ± 7.24	0.82
Metaphase-I*	2.44 ± 2.02	2.49 ± 1.63	2.50 ± 1.59	0.97	2.42 ± 1.22	2.39 ± 1.64	2.59 ± 1.96	0.64
Germinal vesicle oocytes*	1.51 ± 2.10	1.91 ± 2.11	1.57 ± 2.209	0.34	2.06 ± 2.48	1.63 ± 1.97	1.72 ± 2.17	0.57
Atretic oocytes*	0.56 ± 1.01	0.62 ± 1.64	0.72 ± 1.53	0.81	0.87 ± 1.67	0.61 ± 1.55	0.57 ± 1.27	0.56
Rate of fertilization***	82.68	74.73	82.52	0.14	82.52	76.89	80.14	0.61
Total no. of blastocysts*	3.87 ± 2.63	4.36 ± 2.66	4.76 ± 2.65	0.15	4.30 ± 2.87	4.41 ± 2.63	4.20 ± 2.65	0.51
No. of blastocysts vitrified*	3.65 ± 2.53	4.23 ± 3.07	4.59 ± 2.82	0.11	3.81 ± 2.69	4.35 ± 2.80	4 ± 3.01	0.46
No. of blastocyst transferred*	1.54 ± 0.64	1.64 ± 0.59	1.69 ± 0.57	0.29	1.54 ± 0.61	1.66 ± 0.60	1.60 ± 0.60	0.51
Clinical pregnancy rate**	51 (57.95)	99 (67.35)	51 (71.83)	0.15	21 (63.64)	97 (65.54)	83 (66.40)	0.95
Implantation rate***	39.20	45.91	47.87	0.29	50	43.24	44.4	0.65
Live birth rate**	43 (48.86)	84 (57.14)	47 (66.20)	0.25	20 (60.61)	80 (54.05)	74 (59.20)	0.43
*Data presented as Mean ± SD, One-way analysis of variance (ANOVA). **Data presented as n (%), Chi-square test. ***Data presented as percentages. IVF: In vitro fertilization, FSHR: Follicle-stimulating hormone receptor, LHCGR: Luteinizing hormone/choriogonadotropin receptor, N/N: Asn/Asn, N/S: Asn/Ser, S/S: Ser/Ser, BMI: Body mass index, rFSH: Recombinant follicle-stimulating hormone, rHMG: Human menopausal gonadotropin, AFC: Antral follicle count. (IQR for GV Oocytes: 3, Atretic Oocytes: 1)								

**Table 2:** Two-way ANOVA for clinical and IVF outcomes with respect to *FSHR680* and *LHCGR312* variants


***LHCGR312 *** **NN**			
***FSHR680***	**N/N (n = 13)**	**N/S (n = 12)**	**S/S (n = 8)**
Female age (yr)*	29.46 ± 2.82	30.08 ± 2.47	30.38 ± 3.54
Marital life (yr)*	4.76 ± 2.35	5.41 ± 2.31	5 ± 2.00
BMI (kg/m ${\;}^{2}$ )*	25.15 ± 1.77	25.92 ± 1.62	25.88 ± 0.99
Infertility primary**	9 (69.23)	8 (66.67)	6 (75)
Infertility secondary**	4 (30.77)	4 (33.33)	2 (25)
AFC*	19.15 ± 7.85	17.92 ± 8.33	21.63 ± 12.82
IVF failures*	1.4 ± 0.52	1.69 ± 0.94	1.89 ± 1.68
rFSH dose/day (IU)*	219.20 ± 11	222.90 ± 7.2	290.60 ± 26.50
Total rFSH dose (IU)*	2758 ± 520	3031 ± 764	4013 ± 391
Total rHMG dose (IU) (n = 125)*	0.00 ± 0.00	0.00 ± 0.00	0.00 ± 0.00
Total gonadotropin (IU)*	2758 ± 520	3031 ± 764	4013 ± 391
Antagonist (mean No. of days)*	5.53 ± 0.87	5.75 ± 0.75	6.00 ± 0.75
Mean No. of stimulation days*	12.08 ± 1.12	12.67 ± 1.78	13.50 ± 1.20
No. of oocytes*	15.38 ± 7.70	15.08 ± 8.18	17.88 ± 12.86
Metaphase-II*	10.69 ± 5.85	9.83 ± 6.69	10.75 ± 7.63
Metaphase-I*	2.53 ± 0.96	2.16 ± 1.11	2.62 ± 1.76
Germinal vesicle oocytes*	1.53 ± 1.98	2.25 ± 2.56	2.62 ± 3.2
Atretic oocytes*	0.61 ± 0.96	0.66 ± 1.23	1.62 ± 2.82
Rate of fertilization***	87.77	77.42	81.63
Total no. of blastocysts*	4.30 ± 3.37	4.58 ± 2.99	3.87 ± 1.95
No. of blastocysts vitrified*	3.92 ± 3.42	4.00 ± 2.48	3.37 ± 1.76
No. of blastocyst transferred*	1.61 ± 0.50	1.50 ± 0.52	1.50 ± 0.92
Clinical pregnancy rate**	7 (53.85)	8 (66.66)	5 (62.50)
Implantation rate***	30.77	66.67	56.25
Live birth rate**	7 (53.85)	8 (66.67)	5 (62.50)
***LHCGR312 *** **NS** * *			
***FSHR680***	**N/N (n = 33)**	**N/S (n = 84)**	**S/S (n = 31)**
Female age (yr)*	30.42 ± 2.70	30 ± 2.49	31.06 ± 2.93
Marital life (yr)*	5.36 ± 2.59	5.71 ± 2.64	6.12 ± 3.79
BMI (kg/m ${\;}^{2}$ )*	25.39 ± 0.97	25.52 ± 1.46	25.13 ± 0.92
Infertility primary**	29 (87.88)	62 (73.81)	23 (74.19)
Infertility secondary**	4 (12.12)	22 (26.19)	8 (25.81)
AFC*	17.73 ± 7.63	19.17 ± 9.06	16.29 ± 6.42
IVF failures*	1.69 ± 0.94	2.0 ± 1.8	2.2 ± 0.99
rFSH dose/day (IU)*	225.0 ± 15.3	225.0 ± 13.4	284.70 ± 32.10
Total rFSH dose (IU)*	2700 ± 333	2713 ± 389	3610 ± 611
Total rHMG dose (IU) (n = 125)*	0.00 ± 0.00	0.00 ± 0.00	0.00 ± 0.00
Total gonadotropin (IU)*	2700 ± 333	2713 ± 389	3610 ± 611
Antagonist (mean No. of days)*	5.57 ± 0.70	5.59 ± 0.86	5.61 ± 0.98
Mean No. of stimulation days*	11.85 ± 1.12	11.94 ± 1.38	12.48 ± 1.77
No. of oocytes*	14.18 ± 7.55	15.21 ± 8.68	12.77 ± 6.65
Metaphase-II*	9.60 ± 5.53	10.08 ± 6.43	9.03 ± 4.97
Metaphase-I*	2.60 ± 2.29	2.40 ± 1.48	2.16 ± 1.21
Germinal vesicle oocytes*	1.39 ± 1.88	2.00 ± 2.15	0.90 ± 1.19
Atretic oocytes*	0.54 ± 0.83	0.64 ± 1.84	0.61 ± 1.28
Rate of fertilization***	79.73	74.82	79.48
Total no. of blastocysts*	4.30 ± 2.67	4.51 ± 2.77	4.29 ± 2.23
No. of blastocysts vitrified*	4.21 ± 2.48	4.35 ± 2.89	4.51 ± 2.96
No. of blastocyst transferred*	1.66 ± 0.59	1.66 ± 0.60	1.64 ± 0.60
Clinical pregnancy rate**	20 (60.61)	56 (66.67)	21 (67.74)
Implantation rate***	40.91	44.64	41.94
Live birth rate**	15 (45.45)	45 (53.57)	20 (64.52)
Female age (yr)*	30.05 ± 2.47	29.84 ± 3.00	29.91 ± 2.75	0.73
Marital life (yr)*	5.04 ± 2.51	5.92 ± 2.87	5.59 ± 3.22	0.94
BMI (kg/m ${\;}^{2}$ )*	25.33 ± 1.32	25.73 ± 1.22	25.94 ± 1.19	0.28
Infertility primary**	37 (88.10)	43 (84.31)	27 (84.38)	
Infertility secondary**	5 (11.90)	8 (15.69)	5 (15.62)	0.08
AFC*	16.95 ± 7.12	19.31 ± 10.41	20.97 ± 11.52		0.33
IVF failures*	1.34 ± 1.00	1.16 ± 0.89	2.28 ± 1.30	0.24
rFSH dose/day (IU)*	231.50 ± 22.8	226.50 ± 16.1	282.80 ± 33.3	0.39
Total rFSH dose (IU)*	2829 ± 508	2672 ± 324	3402 ± 497	0.02
Total rHMG dose (IU) (n = 125)*	919.6 ± 119.50	880.90 ± 73.2	892.20 ± 101.50	NA
Total gonadotropin (IU)*	3748.81 ± 603.47	3552.45 ± 381.32	4294.53 ± 541.90	0.03
Antagonist (mean No. of days)*	5.38 ± 0.88	5.52 ± 0.70	5.53 ± 0.91	0.89
Mean No. of stimulation days*	12.19 ± 1.55	11.78 ± 0.94	12.06 ± 1.41	0.17
No. of oocytes*	12.67 ± 7.17	15.47 ± 10.50	16.72 ± 11.05	0.41
Metaphase-II*	8.33 ± 4.87	10.66 ± 7.8	11.68 ± 8.4	0.43
Metaphase-I*	2.28 ± 2.06	2.72 ± 1.95	2.78 ± 1.84	0.51
Germinal vesicle oocytes*	1.59 ± 2.33	1.68 ± 1.96	1.96 ± 2.30	0.21
Atretic oocytes*	0.57 ± 1.17	0.56 ± 1.36	0.59 ± 1.29	0.66
Rate of fertilization***	83.43	73.94	85.69	0.95
Total no. of blastocysts*	3.40 ± 2.31	4.07 ± 2.41	5.43 ± 3.04	0.34
No. of blastocysts vitrified*	3.14 ± 2.20	4.09 ± 3.49	4.96 ± 2.87	0.42
No. of blastocyst transferred*	1.42 ± 0.70	1.62 ± 0.599	1.78 ± 0.42	0.34
Clinical pregnancy rate**	24 (57.14)	34 (66.67)	25 (78.12)	0.27
Implantation rate***	40.48	43.14	51.56	0.29
Live birth rate**	21 (50.00)	31 (60.78)	22 (68.75)	0.25
*Data presented as Mean ± SD, ANOVA. **Data presented as n (%), Chi-square test. ***Data presented as percentages, IVF: Invitro fertilization, FSHR: Follicle-stimulating hormone receptor, LHCGR: Luteinizing hormone/choriogonadotropin receptor, N/N: Asn/Asn, N/S: Asn/Ser, S/S: Ser/Ser, BMI: Body mass index, AFC: Antral follicle count, rFSH: Recombinant follicle-stimulating hormone, rHMG: Human menopausal gonadotropin, (IQR for GV oocytes: 3, Atretic oocytes: 1)				

## 4. Discussion 

In this study, we investigated the relationship between *FSHR680* and *LHCGR312* genotypes on the response to controlled ovarian stimulation and clinical outcomes in women undergoing in vitro fertilization in freeze-all cycles. G protein-coupled receptors, *FSHR* and *LHCGR*, are activated by their receptive hormones, FSH and LH (16). These receptors express on their target cells and signal the follicular development through the cAMP/protein kinase A pathway (17). These 2 receptors play an essential role in reproductive biology, and variants in these receptors can produce altered physiological and signaling mechanisms, thus resulting in unsuccessful pregnancy (14, 18). In the present study, we identified a significant difference in the total dose of rFSH between the different genotype groups of *FSHR680* and *LHCGR312* (p = 0.02).

The results indicate that the total rFSH was significantly higher in the SS genotype group of *FSHR680* and the NN genotype group of *LHCGR312* in antagonist cycles (Table II). These findings suggest that women with *FSHR60* SS and *LHCGR312* NN require a higher rFSH dose than other genotype combinations (Table II). Women with homozygous SS in *LHCGR312* were supplemented with LH in the form of hMG, along with an rFSH dose. The total dose of gonadotropins (rFSH+hMG) was higher in the SS genotype group for both *FSHR680* and *LHCGR312* compared to the NN and NS groups. These findings suggest that individuals with the SS genotype of *FSHR* and *LHCGR* may require higher doses of gonadotropins in antagonist cycles, in line with previous studies that have reported a similar association between the 2 genotypes (14).

The reason for supplementation of LH in homozygous for SS in *LHCGR312* was due to poor ovarian response and higher IVF failure in previous cycles performed elsewhere, and observations from previous studies also suggested the addition of LH instead of increasing the FSH dose (7). Even though the supplementation of LH has resulted in a smaller number of oocytes in *LHCGR312* SS, the proportion of GV and atretic oocytes were reduced in this group compared to other *LHCGR312* genotypes generating equal mean number of mature oocytes. These results suggest that LH supplementation may particularly benefit individuals with the *LHCGR312* SS genotype undergoing controlled ovarian stimulation.

Despite LH supplementation in the *LHCGR312* homozygous serine group, the total number of blastocysts formed was comparatively lower than that of the N312 homozygous, though statistically insignificant (Table I). The two-way ANOVA reveals an increasing trend in the mean number of blastocysts formed when combining *LHCGR312 *SS with *FSHR680* genotypes; these results suggest that SS in each polymorphism was advantageous in forming blastocysts (Table II). The mean number of Grade-1 blastocysts increased among *LHCGR312* genotypes (Table I), and *LHCGR312* NS and SS showed some interaction with *FSHR680* genotypes with an increasing trend (Table II).

In the present retrospective study, we identified that women homozygous for SS in both polymorphisms have a 15% higher chance of getting pregnant compared with women NN in *FSHR680* and *LHCGR312* in freeze-all cycles. The results were in concordance with previous studies that there is a higher chance of conception in the serine group (7, 14). The results show that the clinical pregnancy and live birth rates vary across different genotypes of both genes, but the association was not statistically significant. For the *FSHR680* and *LHCGR312*, the SS genotype group had the highest clinical pregnancy and live birth rate. However, the p-value for the clinical pregnancy rate was 0.15, indicating a borderline significance. Analyzing *FSHR680* and *LHCGR312* in combination reveals an increasing trend with the rise in number of S alleles in both polymorphisms. This increase might be attributed to physiological changes in the receptors resulting from the formation of hetero/homo dimers and oligomers, as described previously, rather than the individual receptor alone (18, 19). The decreased pregnancy rate in the presence of the Asn group on both receptors also might be due to decreased cAMP activity (15). The trend in *FSHR680* might be due to the increased signaling capacity of the S680 variant in the presence of FSH in the normal physiological condition as described previously (14). One important observation from the current study is that in freeze-all cycles under the absence of hormonal influence (possible superovulation effect on endometrium receptivity), the clinical pregnancy and live birth rates were significantly lower in the 312SS:680NN group compared to the other 2 groups (312SS:680NS, SS). This suggests that even though the 312SS has an added advantage of getting more pregnancy rates as observed by previous studies, the subgroup analysis revealed that the presence of asparagine in the *FSHR680* had a negative effect in the embryological as well as clinical outcome irrespective of hormone dose (FSH and LH). This study proves that these polymorphisms not only play a role in fresh embryo transfer cycles (6, 7, 14) but also play a significant role in freeze-all cycles in the absence of high levels of hormonal influence concerning clinical outcomes.

A Tang et al., meta-analysis stated that the FSH dose and pregnancy rates were not influenced by *FSHR680* polymorphism. However, the present study observed that the total rFSH dose in antagonist protocol and pregnancy rates were dependent on *FSHR* polymorphism and further influenced by the addition of serine in *LHCGR* (20, 21). The current study investigated the benefit of individualizing the treatment options in infertile women to improve the outcome of ART procedures based on *FSHR680* and *LHCGR312* variants. The results show a promising relationship between genetic variants and the requirement of FSH, LH, and clinical outcomes. The current study findings explain the benefit of genotypes, as suggested by previous authors, along with functional biomarkers like AFC (22, 23, 24). However, the studies using these biomarkers were able to understand the variability in ovarian response to some extent (25). These genetic biomarkers help predict the ovarian response better than hormonal and functional biomarkers, leading to successful COS outcomes. Women with adequate AFC and sufficient hormonal levels show poor response during stimulation (26).

The current findings demonstrated a significant strength for a beneficial effect on clinical outcomes. They provided novel insights into the role of *FSHR680* and *LHCGR312* polymorphisms in freeze-all cycles. This study is the first to investigate the impact of these specific genetic variations on reproductive outcomes, in the absence of any detrimental effects caused by ovarian stimulation on the endometrium during the freeze all embryo transfer cycles. However, a weakness of the paper is the need for a comparative analysis of clinical outcomes between fresh and frozen cycles. Overall, this study contributes significantly to the existing literature by elucidating the role of these specific gene polymorphisms in freeze-all cycle.

## 5. Conclusion

Incorporating the pharmacogenomic approach in the COS regimen helps predict the women with adequate ovarian reserves, but shows a poor response during COS. The current study found that women with homozygous serine in *FSHR* polymorphism require, higher doses of rFSH to develop an adequate number of oocytes in the antagonist cycle. Second, in freeze-all cycles, both *FSHR680* and *LHCGR312* polymorphisms significantly influenced the clinical pregnancy and live birth rates, eliminating the impact of hormonal influences due to superovulation. This highlights the importance of consideration of these genetic variations as predictive markers for reproductive outcomes. Integrating genetic biomarkers along with functional and hormonal biomarkers in COS treatment protocols can help to improve the outcomes of assisted reproduction technologies, which can reduce the socioeconomic stress for the infertile couple planning to have offspring.

##  Data availibility

Data supporting the findings of this study are available upon reasonable request from the corresponding author.

##  Author contributions

Jayesh Amin and Naga Sandhya Alle had complete access to all the data in the study and takes responsibility for both the integrity of the data and the accuracy of the data analysis, including the conceptualization and design of the study. Ami Patel, Bansi Prajapathi, and Paresh Makwana were responsible for the acquisition, analysis, and interpretation of the data. Kota Murali Krishna drafted the manuscript. Jaya Prakash and Kota Murali Krishna conducted statistical analysis. All authors contributed to the critical revision of the manuscript for significant intellectual content. Ami Patel and Kota Murali Krishna took care of the overall coordination and follow-up of the study.

##  Conflict of Interest

The authors declare that there is no conflict of interest.
